# Direct activation of platelets by addition of CaCl_2_ leads coagulation of platelet-rich plasma

**DOI:** 10.1186/s40729-018-0134-6

**Published:** 2018-08-01

**Authors:** Toshihisa Toyoda, Kazushige Isobe, Tetsuhiro Tsujino, Yasuo Koyata, Fumitaka Ohyagi, Taisuke Watanabe, Masayuki Nakamura, Yutaka Kitamura, Hajime Okudera, Koh Nakata, Tomoyuki Kawase

**Affiliations:** 1 Tokyo Plastic Dental Society, Kita-ku, Tokyo, Japan; 2 Bioscience Medical Research Center, Niigata University Medical and Dental Hospital, Niigata, Japan; 3 Division of Oral Bioengineering, Institute of Medicine and Dentistry, Niigata University, Niigata, Japan

**Keywords:** Platelet, Activation, Coagulation, Fibrin, Flow cytometry, Calcium

## Abstract

**Background:**

Based on the notion that full activation of platelets is required for a growth factor release, in regenerative dentistry, platelet-rich plasma (PRP) in liquid form is usually clotted by addition of CaCl_2_ in glassware before topical implantation. However, there has been no evidence as to which is better, full or partial activation of platelets, for minimizing the loss of growth factors and improving the controlled release of growth factors from coagulated PRP. To address this matter, here, we primarily examined direct effects of CaCl_2_ on platelets in PBS and on coagulation in citrated PRP.

**Methods:**

PRP was prepared from healthy volunteers’ blood. Platelets’ actions were monitored by scanning electron microscopy, flow cytometry, digital holographic microscopy, and immunofluorescent staining. Clot formation was examined in plasma.

**Results:**

In plasma-free PBS, 0.1% CaCl_2_ immediately upregulated CD62P and CD63, causing a release of microparticles and fibrinogen/fibrin; consequently, platelets aggregated and adhered to polystyrene culture dishes with enlargement of their attachment area. In a clot formation assay in plasma, CaCl_2_ initially induced platelet aggregation, which triggered loop-like matrix formation and subsequently induced coagulation on a watch glass. Such changes were not clearly observed either with PRP in a plastic dish or in platelet-poor plasma on a watch glass: coagulation was delayed in both conditions.

**Conclusions:**

These findings indicate that besides the well-known coagulation pathway, which activates platelets via thrombin conversion in a coagulation cascade, CaCl_2_ directly activates platelets, which then facilitate clot formation independently and in cooperation with the coagulation pathway.

## Background

Since Marx’s report [1], platelet-rich plasma (PRP) and subsequently modified PRP derivatives have been widely applied in regenerative dentistry. Unlike self-clotted platelet-rich fibrin (PRF), for better handling efficiency and minimizing the loss of growth factors to diffusion, PRP and some other derivatives in liquid form are usually clotted by addition of exogenous coagulation factors, such as thrombin and/or CaCl_2_. For example, in the case of plasma-rich in growth factors (PRGF) (the most successful PRP derivative) [2], venipuncture is performed with anticoagulants, usually citrate or acid citrate dextrose (ACD), to chelate plasma Ca^2+^ [3]. Somewhat excessive amounts of Ca^2+^ are recommended for addition to citrated PRGF preparations to reconstitute plasma by recovering free Ca^2+^ levels on a watch glass at 37 °C [4, 5].

Behind this clot formation, there is the intrinsic coagulation pathway, which is activated at the level of factor XII by the glass surface and proceeds in the presence of Ca^2+^ to convert prothrombin to thrombin, subsequently fibrinogen to fibrin, and consequently facilitates fibrin polymerization and cross-linking [6]. In this process, thrombin converted from prothrombin is known to activate platelets via specific subtypes of protease-activated receptors [7, 8]. Therefore, it is likely that added CaCl_2_ indirectly activates platelets through activation of a coagulation pathway in citrated PRP in glassware. The resulting fibrin fibers are thick and well cross-linked and are almost identical to those formed in a preparation of PRF [9].

In contrast to Ca^2+^, when an alternative coagulation factor, e.g., thrombin, is added to citrated PRP, the resulting fibrin fibers are thin and often fused together turning into a sheet-like matrix. Because this thrombin-induced fibrin matrix is relatively easily degradable [9], to stabilize its existence at an implantation site and to retain its growth factors longer, it is better to use Ca^2+^ as a coagulation factor and to employ glassware for activation of the intrinsic coagulation pathway. This approach is not limited to PRP and PRGF and can be extended to PRF preparation from stored whole-blood samples. Nonetheless, direct effects of exogenously added Ca^2+^ on platelets in vitro have been poorly investigated and understood.

In this study, we attempted to dissociate platelets from the coagulation pathway and to evaluate possible direct action of Ca^2+^ on platelet functions in citrated whole-blood samples. In addition, in response to recent increasingly frequent requests for scheduled or outsourced, but not immediate on-site, preparation of various platelet concentrate types [4, 5], we examined time course changes in platelet responsiveness to added CaCl_2_ along with coagulation time in stored whole-blood samples.

## Methods

### Preparation of the PRP fraction and clotting

Blood samples were collected from eight nonsmoking healthy male volunteers at ages from 32 to 68 years. The study design and consent forms of all the procedures performed were approved by the ethics committee for human participants of the Niigata University School of Medicine (Niigata, Japan) in accordance with the Helsinki Declaration of 1964 as revised in 2013.

Peripheral blood (~ 9 mL) was collected into plastic vacuum plain blood collection tubes (Neotube; NIPRO, Osaka, Japan) containing 1 mL of the A-formulation of ACD (ACD-A; Terumo, Tokyo, Japan) and was immediately centrifuged at 530×*g* for 10 min. The upper plasma fraction was collected, transferred to fresh tubes, and served as a PRP fraction [10]. The numbers of platelets and other blood cells in whole-blood samples and PRP preparations were determined on an automated hematology analyzer (pocH 100iV, Sysmex, Kobe, Japan).

Evaluation of platelet surface antigen expression by immunofluorescence (IT) staining

Platelet concentrates were prepared from citrated whole-blood samples, pretreated with 5 μg/mL prostaglandin E_1_ (PGE_1_; Cayman Chemical Co., Ann Arbor, MI, USA) for 5 min, rinsed, and resuspended in PBS in sample tubes. Washed platelets were then treated with CaCl_2_ at a final concentration of 0.1% and incubated in polystyrene culture dishes for up to 30 min at ambient temperature. At the end of the incubation, the reaction was stopped by adding 10% of neutralized formaldehyde. Platelets were washed twice, blocked with 0.1% Block-Ace (Sumitomo Dainippon Pharma Co., Ltd., Osaka, Japan) in 0.1% Tween 20-containing Tris-buffered saline (TBS) (T-TBS) for 1 h, and treated with a mouse monoclonal anti-human CD41, anti-CD62P, or anti-CD63 antibody (1:20 dilution; BioLegend, San Diego, CA, USA) overnight at 4 °C. At the end of treatment, the platelets were again washed twice with T-TBS and were then probed with a secondary antibody, i.e., a goat anti-mouse IgG H&L antibody (conjugated with Alexa Flour 555; 1:50 dilution; Abcam, Cambridge, MA, USA), for 30 min at ambient temperature. Finally, after a subsequent PBS wash, the platelets were mounted with an antifade mounting medium (Vectashield; Vector laboratories, Burlingame, CA, USA), and CD41, CD62P, and CD63 expression levels were examined under a fluorescence microscope equipped with a cooled charge-coupled device (CCD) camera (Nikon, Tokyo, Japan).

### Flow cytometric analysis

As described in the subsection above, the platelet fractions were prepared and treated with CaCl_2_ in polypropylene sample tubes. At the end of incubation, the platelets were fixed with an equal volume of a commercial fixative, ThromboFix (Beckman-Coulter, Brea, CA, USA), for 30 min, washed twice with PBS, and probed simultaneously with phycoerythrin (PE)-conjugated mouse monoclonal anti-CD41 and FITC-conjugated anti-CD62P or FITC-conjugated CD63 antibodies (5 μL per 100 μL of a sample) (BioLegend) for 40 min at ambient temperature. After two washes with PBS, the platelets were analyzed on a flow cytometer (Cell Lab Quanta SC; Beckman-Coulter Inc., Brea, CA, USA) as described before [5]. For isotype controls, mouse IgG1 (BioLegend) was employed. The data were analyzed in the FlowJo software (FlowJo, LLC, Ashland, Oregon, USA).

### Scanning electron microscopy (SEM)

As described above, to observe changes in platelet appearance, washed platelets were treated with 0.1% CaCl_2_ in polypropylene sample tubes for up to 20 min and placed in polystyrene cell culture dishes where they were incubated for the last 5 min of treatment. Alternatively, to examine microparticles and platelet-derived fibrin fibers, washed platelets were treated with 0.1% CaCl_2_ in polypropylene sample tubes, were immediately transferred onto specific filters (Sem Pore; JOEL, Akishima, Japan), and incubated for up to 15 min. At the end of treatment, the platelets were washed with PBS, fixed with 2.5% neutralized glutaraldehyde, serially dehydrated in ethanol and *t*-butanol solutions, and freeze-dried.

Individual fibrin clots were compressed in a stainless steel compressor (JMR, Niigata, Japan) to eliminate abundant serum proteins with the exudate [11], washed three times with PBS, fixed with 2.5% neutralized glutaraldehyde, serially dehydrated in ethanol and *t*-butanol solutions, and freeze-dried.

After that, these samples were examined under a scanning electron microscope (TM-1000; Hitachi, Tokyo, Japan) at an accelerating voltage of 15 kV, as described previously [9].

### Quantitative assessment of cell morphology by digital holographic microscopy (DHM)

As described in the section on SEM examination of platelets in cell culture dishes, washed platelets were treated with 0.1% Ca in sample tubes and plated in cell culture dishes (or in flasks) where they were incubated for the last 5 min of treatment. After fixation with 2.5% neutralized glutaraldehyde, the platelets were washed with PBS and stored in PBS until DHM examination.

Imaging by DHM (HoloMonitor M4; Phase Holographic Imaging AB, Lund, Sweden) was performed as described elsewhere [12]. The data were analyzed using specialized software, HoloStudio M4 (Phase Holographic Imaging AB). For surface roughness and area analysis, after a series of images were captured, the grayscale images were converted to the black-and-white format by the Otsu method, and the cell identification and segmentation in the images were adjusted either automatically or manually.

Based on the accumulated data on the cell refractive index, the average refractive index for cultured monolayer cells was fixed at 1.38 (Phase Holographic Imaging AB, *personal communication*). This value was applied to platelets. The refractive index of the surrounding medium is 1.34 and should not excessively deviate (± 0.08) from the cell refractive index.

On the basis of our preliminary data, we focused on the parameters related to the cell area and roughness and examined at least 4000–8000 platelets in each platelet population.

### Determination of prothrombin time

This parameter was determined by means of a Coaguchek XS system (Roche Diagnostics International Ltd., Basel, Switzerland). For citrated samples, 500 μL of whole-blood samples was pre-warmed at 37 °C for 5 min, mixed well with 10 μL of 10% CaCl_2_ by gentle inverting, and incubated for 5 min prior to the analysis.

### A clot formation assay

Next, PRP fractions were centrifuged at 1060×*g* for 5 min to fractionate platelet-poor plasma (PPP). PRP or PPP (1.5 mL) was mixed with 10% CaCl_2_ in the ratio mentioned for whole-blood samples on a watch glass or in a plastic dish. To activate platelets, small cut pieces of a collagen sponge composed of collagen microfibers (Integran®; Koken, Tokyo, Japan) were added to PRP in the plastic dish. Time for complete clot formation was determined.

### Statistical analysis

The data were expressed as mean ± standard deviation (SD). For multigroup comparisons, statistical analyses were conducted to compare the mean values by one-way analysis of variance (ANOVA), followed by Dunn’s multiple-comparison test (SigmaPlot 12.5; Systat Software, Inc., San Jose, CA, USA). Differences with *P* values of < 0.05 were considered statistically significant.

## Results

**Fig. 1:**
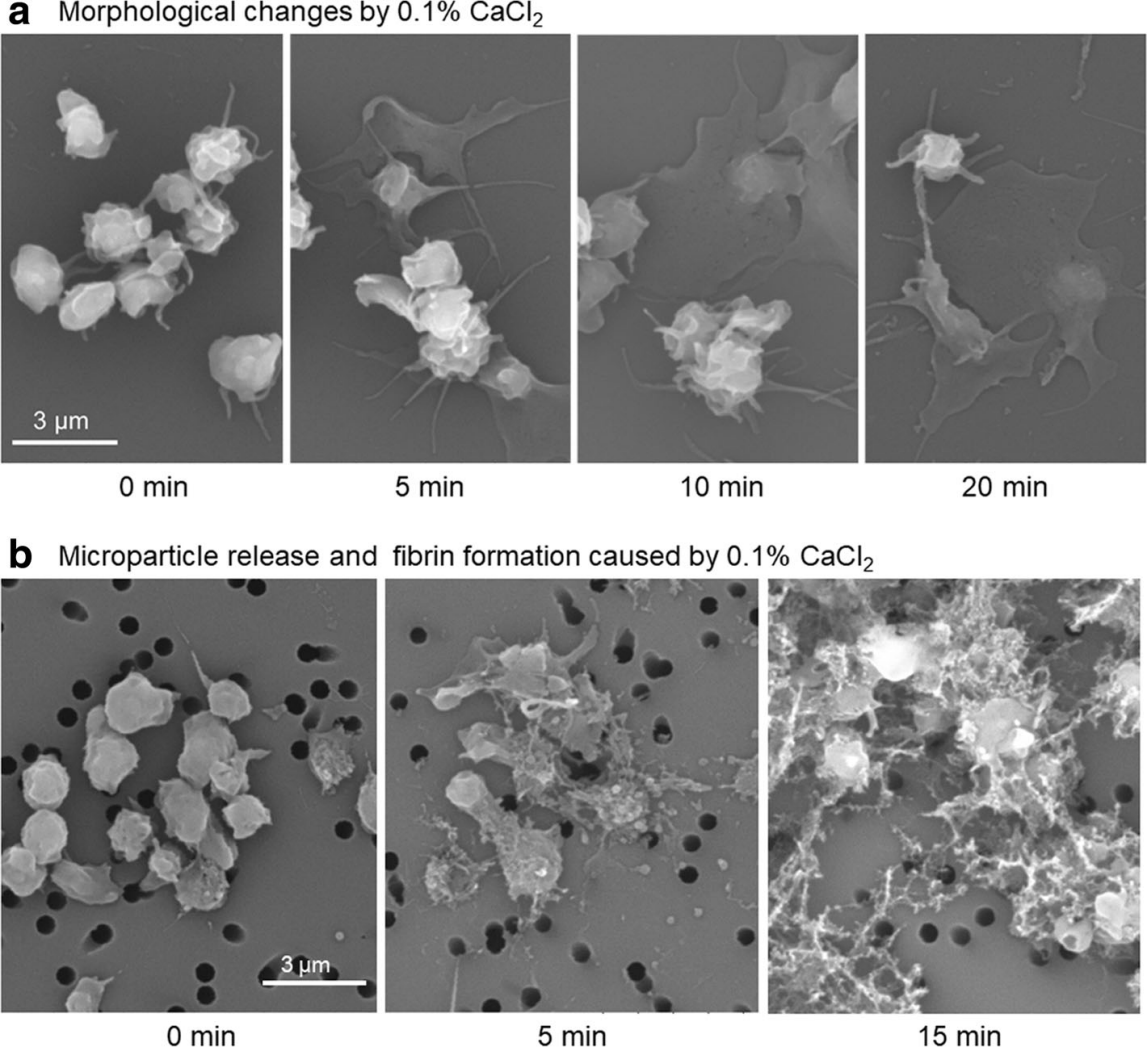
Morphological changes of (and microparticle release and fibrin formation by) Ca^2+^-stimulated platelets. **a** Washed platelets were treated with 0.1% CaCl_2_ in sample tubes and then in culture dishes for 5, 10, and 20 min in total. **b** Washed platelets were treated with 0.1% CaCl_2_ in sample tubes, immediately transferred onto filters, and incubated for 5 and 15 min in total. Control platelets were directly placed in culture dishes or filters and fixed. Similar results were obtained from samples of three other donors

Morphological changes of (and microparticle release and fibrin formation by) Ca^2+^-stimulated platelets are shown in Fig. 1. When washed platelets were treated with 0.1% CaCl_2_ in sample tubes and plated in culture dishes for the last 5 min of treatment (control, 2 min), platelets’ ability to adhere to the dish bottom surface increased with the duration of Ca^2+^ treatment (Fig. 1a). On the other hand, when washed platelets were treated with 0.1% CaCl_2_ in sample tubes, immediately transferred onto percolated filters, and incubated for up to 15 min, the platelets were found to release microparticles and fibrinogen, which was converted to fibrin even in the absence of plasma components (Fig. 1b).

**Fig. 2:**
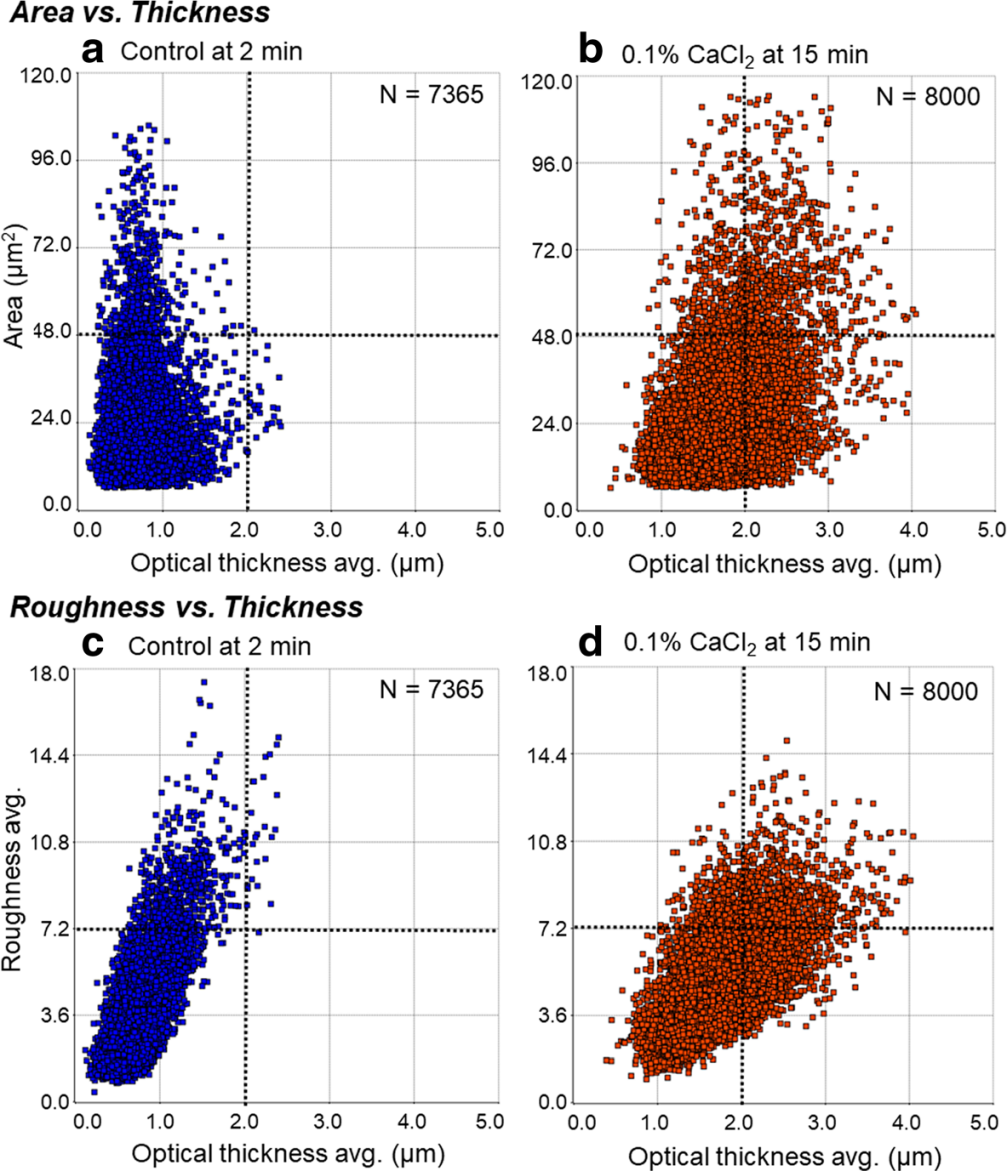
DHM examination of changes in attachment area, optical thickness, and surface roughness of Ca^2+^-simulated platelets. Washed platelets were treated with 0.1% CaCl_2_ for 15 min (**b**, **d**) on culture flasks, fixed, and subjected to DHM examination. Control was no treatment at 2 min (**a**, **c**). Each platelet was plotted in the scatter plots of “Area vs. Thickness” (**a**, **b**) or “Roughness vs. Thickness”. Similar results were obtained from samples of two other donors

Changes in attachment area, optical thickness on average, and surface roughness of Ca^2+^-simulated platelets are shown in Fig. 2. In the control, i.e., resting platelets, the average thickness of almost all platelets was within 2 μm. Among the platelets stimulated by 0.1% Ca^2+^ for 15 min, approximately 50% of the cells increased their apparent thickness beyond 2 μm. Attachment area was also significantly enlarged by added Ca^2+^, but roughness was not changed. In this case, enlarged platelets in terms of both thickness and area definitely represent aggregated platelets, but, probably, thicker platelets also represent aggregated platelets.

**Fig. 3:**
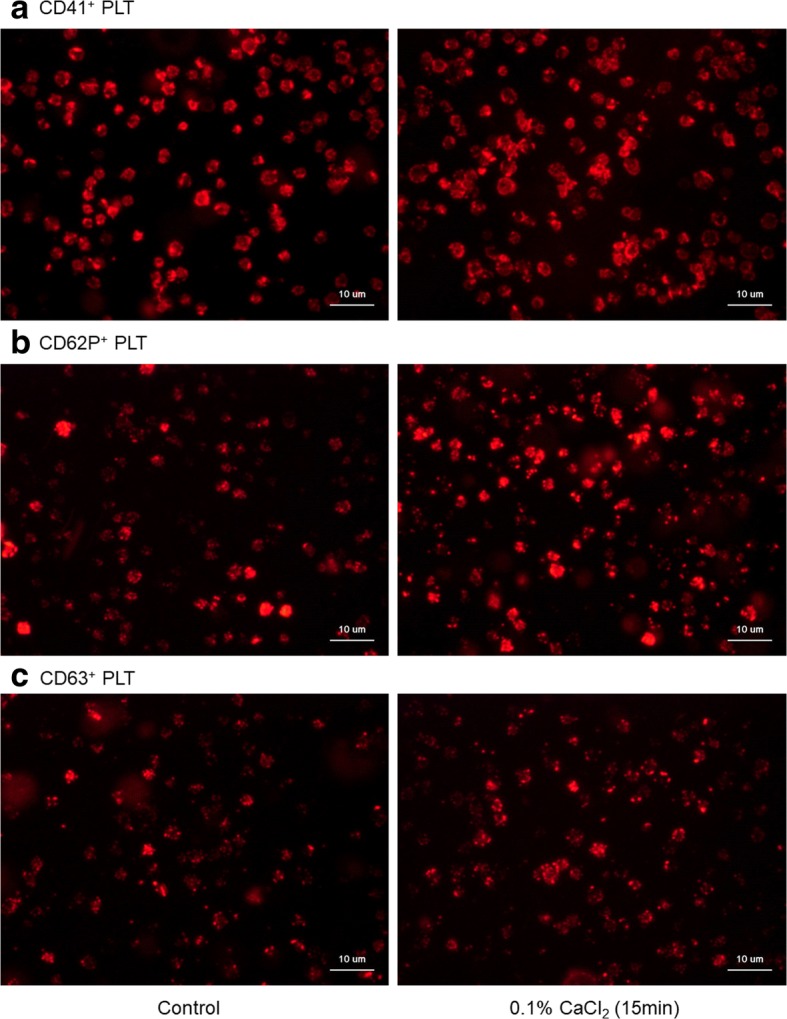
Immunofluorescent (IF) evaluation of changes in surface marker expression—CD41 (**a**), CD62P (**b**), and CD63 (**c**)—in Ca^2+^-stimulated platelets. PLT platelets. Washed platelets were treated with 0.1% CaCl_2_ in culture dishes and subjected to IF staining. Similar results were obtained from samples of three other donors

Immunofluorescent (IF) evaluation of changes in surface marker expression in Ca^2+^-stimulated platelets is shown in Fig. 3. When washed platelets were treated with 0.1% CaCl_2_ in sample tubes, immediately placed in culture dishes, and incubated for 15 min, CD62P and CD63, but not CD41, were substantially upregulated.

**Fig. 4:**
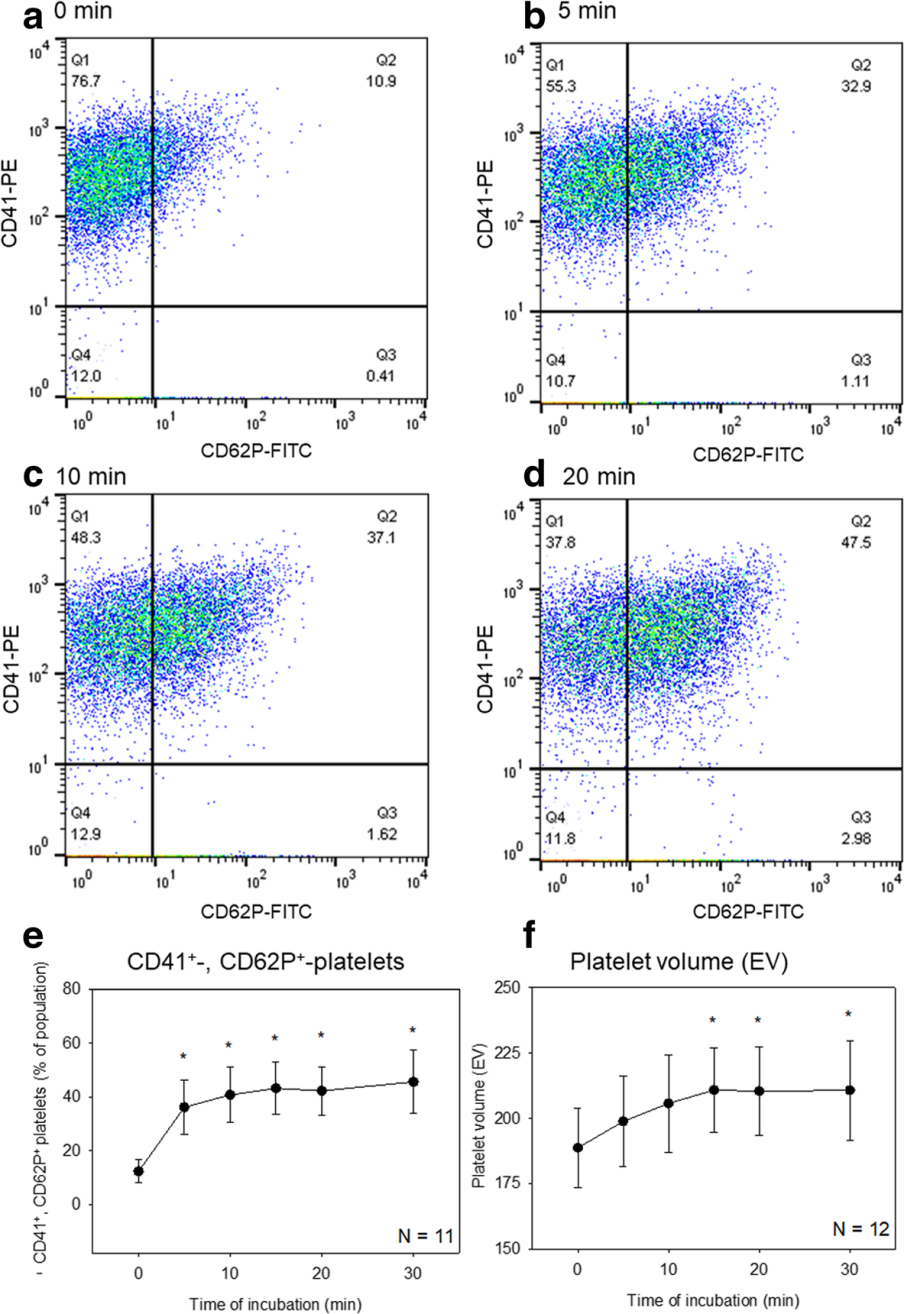
Flow cytometric analysis of changes in surface marker expression in Ca^2+^-stimulated platelets. Washed platelets were prepared and treated as described in the caption of Fig. 1a. **a**–**d** Representative scatter plots of CD41^+^ and CD62P^+^ platelets. **e** Effects of Ca^2+^ on percentages of double-positive platelets in whole platelet populations (*N* = 11). **f** Effects of Ca^2+^ on platelet volume (*N* = 12). Asterisks indicate statistically significant differences between the control data and treatment data

Flow cytometric analysis of changes in surface marker expression in Ca^2+^-stimulated platelets is presented in Fig. 4. When washed platelets were treated with 0.1% CaCl_2_ in sample tubes for up to 30 min, CD62P^+^ platelet counts in CD41^+^ platelet populations increased time-dependently. Similarly, platelet volume, as assessed by impedance, increased with the duration of Ca^2+^ treatment.

**Fig. 5:**
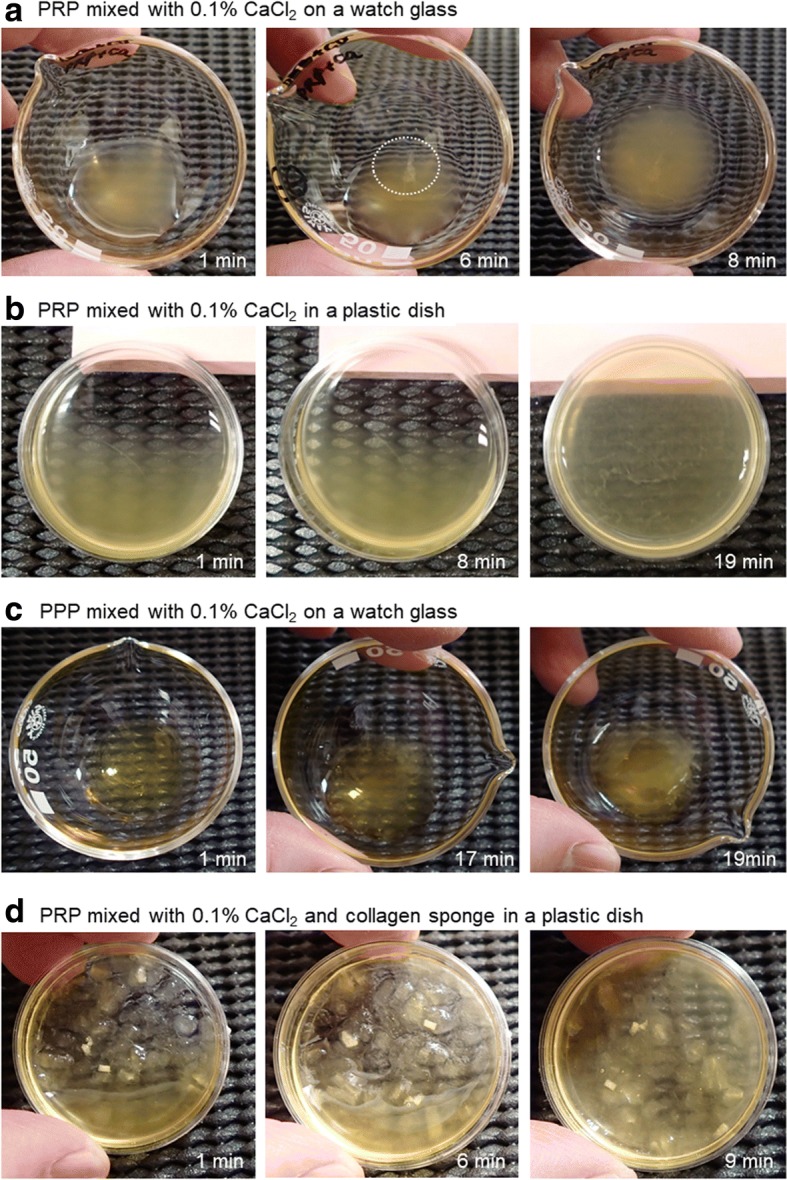
Effects of CaCl_2_ on fibrin clot formation on watch glasses and polystyrene culture dishes. **a** PRP mixed with 0.1% CaCl_2_ on a watch glass. **b** PRP mixed with 0.1% CaCl_2_ in a plastic dish. **c** PPP mixed with 0.1% CaCl_2_ on a watch glass. **d** PRP mixed with 0.1% CaCl_2_ and a collagen sponge in a plastic dish. Similar results were obtained from three other donors

Because these findings taken together implied that exogenously added Ca^2+^ beyond physiological in vivo levels (approximately 9.0 vs. 2.5 mM) directly activates platelets in the absence of plasma components, platelets’ direct involvement in coagulation was then examined using platelet-rich and platelet-poor plasma. Effects of CaCl_2_ on fibrin clot formation on watch glasses and in polystyrene culture dishes are shown in Fig. 5. On watch glasses (panel a, control), addition of 0.1% CaCl_2_ most rapidly formed loop-like substances (in a dashed-line circle) and subsequently fibrin clots in PRP at 8 min of treatment (Fig. 5a). Polystyrene culture dishes, whose surface is optimized for adherent cells, significantly delayed Ca^2+^-induced clot formation in PRP from 8 to 19 min (Fig. 5b). When the treatment was carried out in PPP, clot formation was also significantly delayed (Fig. 5c). Nonetheless, in the presence of the collagen sponge, Ca^2+^ addition caused formation of a fibrin clot in PRP in culture dishes as rapidly as in the control (Fig. 5d vs. a).

**Fig. 6:**
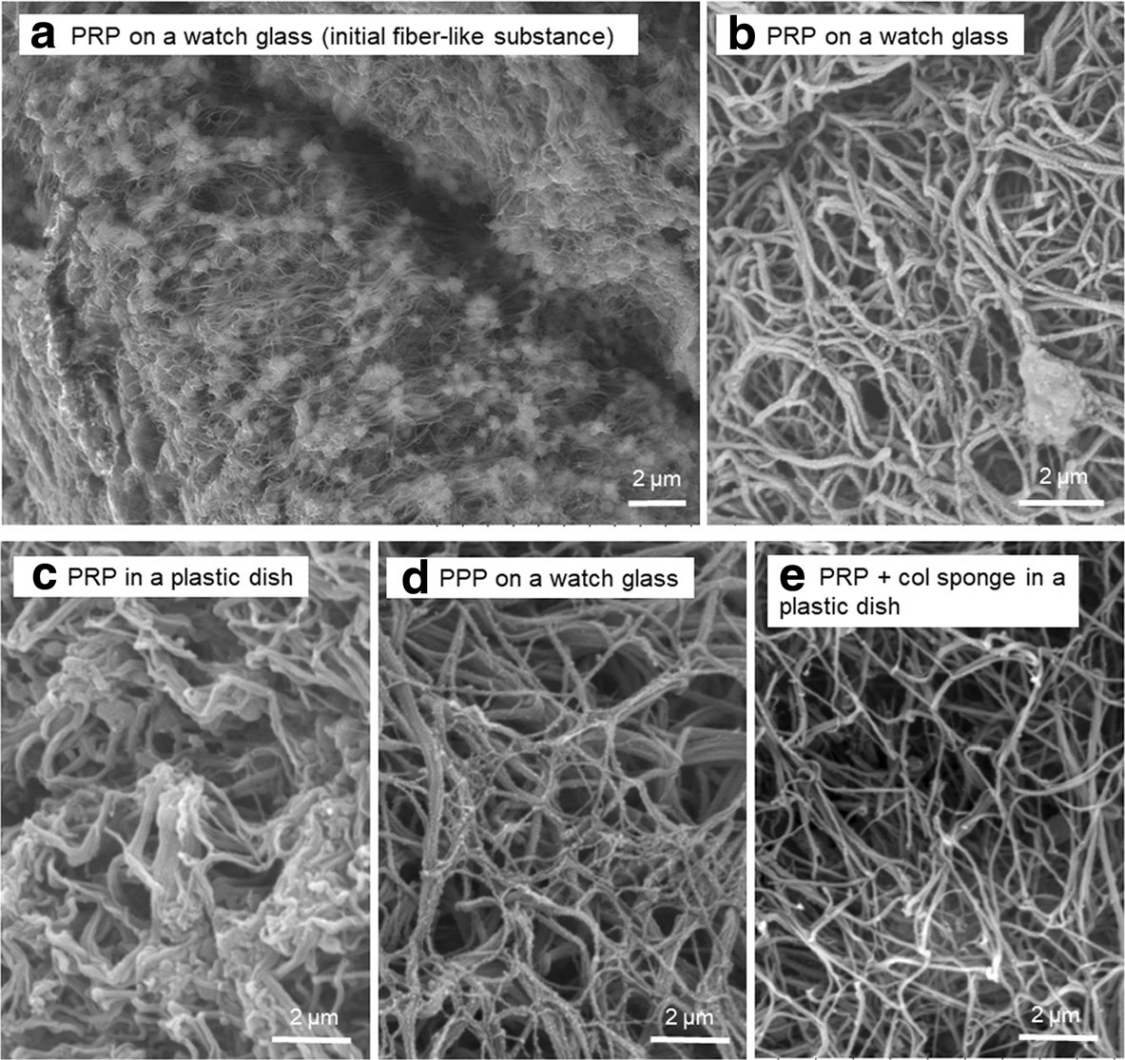
SEM analysis of the surface microstructures of formed fibrin clots. **a** A fiber-like substance initially formed in Ca^2+^-treated PRP on a watch glass. **b** The fibrin clot formed in Ca^2+^-treated PRP on a watch glass. **c** The fibrin clot formed in Ca^2+^-treated PRP in a plastic culture dish. **d** The fibrin clot formed in PPP on a watch glass. **e** The fibrin clot formed in PRP in the presence of a collagen sponge in a plastic dish. Similar results were obtained from three other donors

The surface microstructures of the formed fibrin clots are depicted in Fig. 6. The loop-like substances that initially formed in PRP on watch glasses were composed of abundant aggregated platelets, and relatively smaller amounts of fibrin fibers were deposited around platelet aggregates (Fig. 6a). By contrast, in the final version of fibrin clots, platelet aggregates were hardly detectable and most parts consisted of fibrin fibers (Fig. 6b). Compared with the control clots, those formed in culture dishes were enriched in platelets although either thickness or cross-link density of fibrin fibers was apparently similar to that in the control clots (Fig. 6c). Fibrin clots formed from PPP on watch glasses and from PRP in culture dishes with collagen sponges were similar to the control clots.

**Fig. 7:**
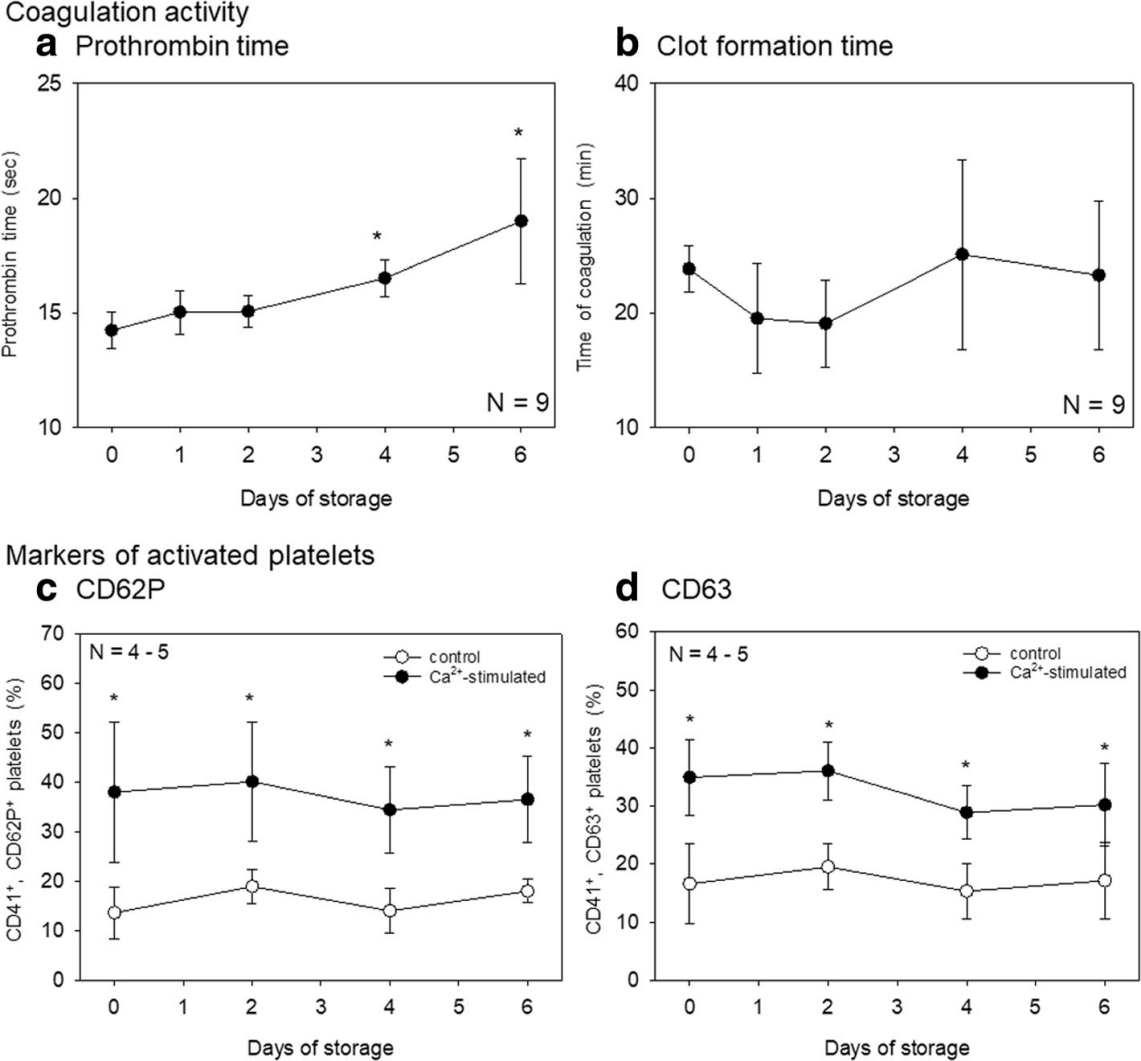
Effects of storage time on the coagulation pathway and platelets. Prothrombin time (**a**) and clot formation time (**b**) of citrated whole-blood samples stored for up to 6 days were examined simultaneously. Platelets’ responsiveness to Ca^2+^ was assessed by upregulation of CD62P (**c**) and CD63 (**d**) in CD41^+^ platelets

Finally, effects of storage time on coagulation activity and platelet functions were examined. Effects of Ca^2+^ on prothrombin time and clot formation of stored whole-blood samples are shown in Fig. 7a, b. Because we preliminarily confirmed that reconstitution of citrated blood with CaCl_2_ can recover the conditions applicable to the prothrombin time assay, we evaluated prothrombin time of citrated whole-blood samples stored for up to 6 days. Prothrombin time increased with the duration of storage. Effects of Ca^2+^ on CD62P and CD63 expression in platelets isolated from citrated whole-blood samples stored for up to 6 days are presented in Fig. 7c, d. The responsiveness to Ca^2+^ in terms of both CD62P and CD63 at 15 min seemed to somewhat decrease with storage time; however, even after 6-day storage, platelets maintained their response to added Ca^2+^: upregulation of CD62P and CD63 (vs. control levels).

## Discussion

It is well known that platelets are activated by adenosine diphosphate (ADP), thrombin, epinephrine, thromboxane A_2_, collagen, and many other compounds and thus aggregate through binding of fibrinogen and glycoprotein IIb/IIIa receptors and upregulate surface antigens known as “platelet activation markers”: CD62P and CD63 [13, 14]. During activation, platelet morphology generally changes from a disc-shaped appearance (resting) to a rolling ball-shaped appearance, hemisphere-shaped appearance, and finally spreading adhesion appearance [15]. To our knowledge, however, increased extracellular free Ca^2+^ levels have not been sufficiently studied as an activator of platelets probably because free Ca^2+^ levels are maintained within some range, but not depleted, under pathological conditions of plasma and tissues.

Compared with the in vivo settings, citrated whole blood provides totally different conditions to platelets: extracellular free Ca^2+^ is chelated by citrate. The primary purpose of addition of Ca^2+^-chelating anticoagulants is to inhibit serial reactions of the coagulation pathway. Nevertheless, it is known that this chelation also suppresses various platelet functions related to aggregation [16]. Platelet activators mentioned above elevate intracellular Ca^2+^ concentrations, either by releasing Ca^2+^ from intracellular stores or by increasing Ca^2+^ influx across the plasma membrane [17]. Conversely, acute chelating of extracellular Ca^2+^ by EGTA inhibits the platelet ability to adhere to glass and to aggregate in response to ADP or other platelet activators [16].

The mechanism of Ca^2+^-induced platelet activation is discussed below. We preliminarily postulated expression of Ca^2+^-sensing receptors (CaSR) but failed to detect this type of receptor by the methods of flow cytometry and IF staining with a mouse monoclonal anti-CaSR antibody (cat. # ab19347; Abcam). No appreciable specific binding was observed in IF staining, and fewer than 5% of platelets tested positive in the flow cytometric analysis. Therefore, because this CaSR antigen is known to be expressed in the brain, kidneys, lungs, liver, heart, skeletal muscle, and placenta [18], but not in platelets, it is reasonable to rule out the participation of CaSR in Ca^2+^-induced platelet activation in our study.

An alternative possibility may be related to the status of platelets stored in a low-Ca^2+^ environment: platelets constantly increase thromboxane A_2_ (TXA_2_) production and tend to easily aggregate under the influence of the TXA_2_ autocrine loop [19]. Therefore, it is thought that platelets prepared from citrated whole-blood samples continuously repeat Ca^2+^ discharge from intracellular Ca^2+^ stores and Ca^2+^ efflux across the plasma membrane [20] in response to endogenously produced TXA_2_. In addition, even though the autocrine loop of TXA_2_ does not function actively, intracellular free Ca^2+^ levels of platelets (~ 100 nM at actual resting levels) are maintained also by a balance between the “passive” leak of Ca^2+^ into platelets and the concurrent efflux of Ca^2+^ across the plasma membrane and accumulation in intracellular stores [21]. Therefore, in a citrated medium, it is plausible that intracellular free Ca^2+^ in platelets is depleted gradually by repeated Ca^2+^ discharge from intracellular stores and passive and active Ca^2+^ flux across the plasma membrane. We can speculate that Ca^2+^ addition may promptly enable Ca^2+^ entry via the Ca^2+^ leak pathway and subsequently activate platelets by the autocrine pathway of TXA_2_ or activators stored in α-granules, such as ADP and thrombin. In support of this possibility, Aoki et al. demonstrated that added Ca^2+^ increases thrombin production by washed platelets [22]. Further investigation is needed to clarify the mechanism of Ca^2+^-induced platelet activation.

The mechanism of Ca^2+^-induced clot formation is worthier of discussion. In the human body, two types of thrombosis are known: white and red thrombi [23]. According to this definition, a fibrin mesh is deposited on platelet aggregates in a white thrombus, whereas platelets (and red blood cells) are trapped and aggregated by the fibrin mesh in a red thrombus. In this study, we found that platelet aggregates function like nuclei of clot formation. Therefore, platelets are located mainly near the center or in a deep region of a clot, and this clot may be classified essentially as a “white thrombus.” In this case, growth factors stored in platelets can be assumed to be retained for a relatively long time. It is possible that this type of clot functions as a long-lasting carrier with a better regenerative potential.

On the other hand, in PRF prepared from fresh whole-blood without anticoagulants, it is thought that the centrifugal force increases the contact of factor XII with a glass surface, thereby primarily forming fibrin clots. Although platelets can also be activated by a glass surface, the centrifugal force accumulates platelets at and just below the interface between the red blood cell fraction and plasma fraction as well as in the surface area of PRF preparations [11]. Even though only a few red blood cells are embedded in the clot, in terms of the formation mechanism, this clot could be classified as a “red thrombus.” Therefore, it is plausible that growth factors that are stored in the platelets trapped in fibrin mesh may be released at early phases of degradation.

As described previously [24], glass tube production is no longer practiced by major manufacturers of medical equipment in Japan and in major Western countries. One of the possible solutions (when glassware is needed) is to coat the inner wall of plastic tubes with micronized silica particles, as in Greiner Bio-One Serum Clot Activator Tubes (Greiner Bio-One North America Inc., Monroe, NC, USA). Likewise, various types of plastic vacuum blood collection tubes containing a coagulation-activating film are produced by several manufacturers. Nevertheless, these products are intended for routine clinical examination, and their quality, especially safety, has never been ensured for preparation and implantation of platelet concentrates.

If platelet activity can be controlled in well-qualified plastic tubes, it is expected that safer PRF will be provided to patients without activation of the intrinsic coagulation pathway. In this study, we demonstrated that a collagen sponge can rapidly facilitate fibrin clot formation (without the aid of glassware) probably via activation of platelets. On the basis of this concept, we recently developed a PRF kit composed of plastic tubes containing a synthetic, RGD motif-enriched collagen-like peptide (RCP; FUJIFILM, Tokyo, Japan) and validated its utility in PRF preparation and its efficacy in bone regeneration [Tsukioka et al., manuscript submitted]. This fibrin clot formation is triggered by activated platelets.

## Conclusions

**Fig. 8:**
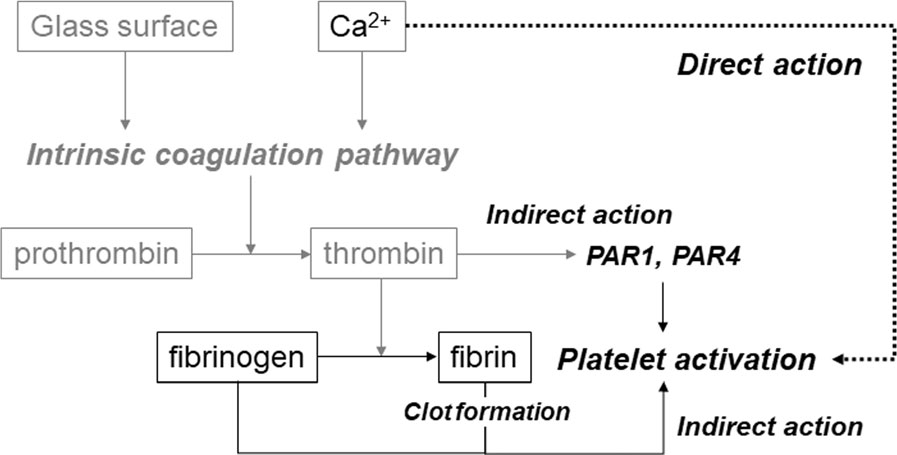
Mechanisms of Ca^2+^-induced fibrin clot formation in citrated plasma. As a major mechanism, Ca^2+^ activates the coagulation pathway in cooperation with a glass surface and thus subsequently activates platelets through production of thrombin and fibrin. As an additional mechanism, Ca^2+^ directly stimulates platelets to promote coagulation

In addition to the well-known intrinsic coagulation pathway, which activates platelets via thrombin conversion, added Ca^2+^ may directly activate washed platelets and promote clot formation alone and in cooperation with the coagulation pathway as illustrated in Fig. 8. Therefore, it is possible to control platelet activation levels and the consequent growth factor release by modulating not only indirect but also direct activation pathways.

## Abbreviations

ACD: Acid citrate dextrose solution
ADP: Adenosine diphosphate
CaSR: Calcium-sensing receptor
DHM: Digital holographic microscopy
PBS: Phosphate-buffered saline
PPP: Platelet-poor plasma
PRF: Platelet-rich fibrin
PRP: Platelet-rich plasma
SEM: Scanning electron microscope
TBS: Tris-buffered saline
TXA_2_: Thromboxane A_2_
